# [Corrigendum] Crocin exerts anti-inflammatory and anti-catabolic effects on rat intervertebral discs by suppressing the activation of JNK

**DOI:** 10.3892/ijmm.2026.5904

**Published:** 2026-06-29

**Authors:** Kang Li, Yan Li, Zhenjiang Ma, Jie Zhao

Int J Mol Med 36: 1291-1299, 2015; DOI: 10.3892/ijmm.2015.2359

Following the publication of the above paper, a concerned reader has drawn to the Editor's attention that, regarding the immunohistochemical images shown in Fig. 6A and B on p. 1297, the 'Control' and 'LPS+Crocin' panels in Fig. 6A showed an overlapping section, such that these data, which were intended to show the results of differently performed experiments, had apparently been derived from the same original source. In addition, the 'LPS+Crocin' panel in Fig. 6B contained data that subsequently reappeared in a paper published by the same research group in the journal *PeerJ*.

The authors were contacted by the Editorial Office to offer an explanation for these apparent anomalies in the presentation of the data in this paper, and the placement of the wrongly identified images in the figure was caused by errors that occurred during the process of copying the multiple images from the microscope system to the computer. The corrected version of Fig. 6, now showing the correct images for the 'Control' and 'LPS+Crocin' data panels in Fig. 6A, and the 'LPS' and 'LPS+Crocin' data panels in Fig. 6B (both the NBT- and DAPI-stained images), is shown on the next page. The authors confirm that the errors associated with this figure did not have any significant impact on either the results or the conclusions reported in this study, and all the authors agree with the publication of this Corrigendum. The authors are grateful to the Editor of *International Journal of Molecular Medicine* for allowing them the opportunity to publish this Corrigendum; furthermore, they apologize to the readership of the Journal for any inconvenience caused.

## Figures and Tables

**Figure 6:**
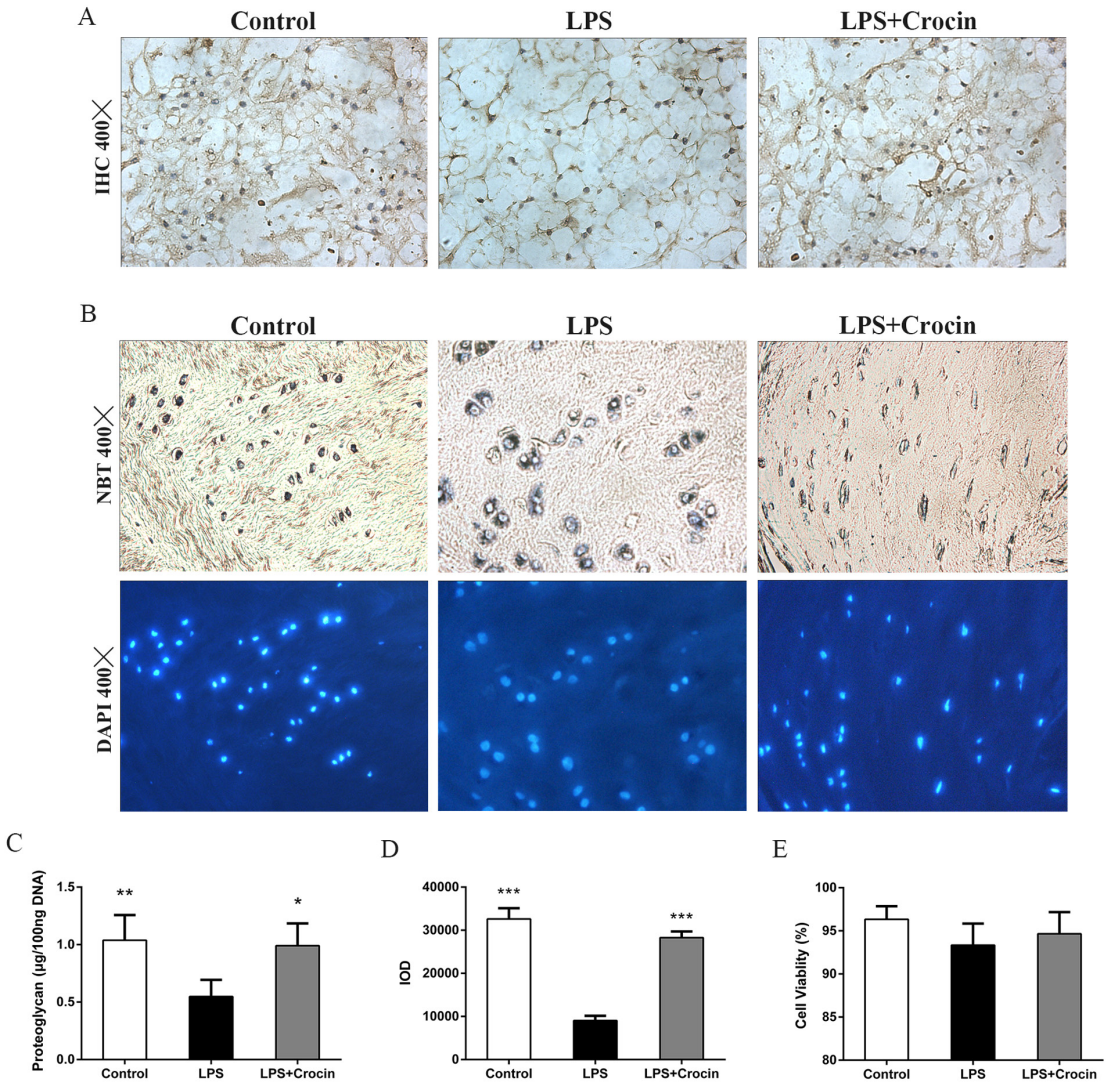
Immunohistochemical, DMM B and cell viability assays of intervertebral discs (IVDs) cultured *ex vivo*. (A) Collagen-II expression in IVDs (magnification, ×400). (B) NBT- and DAPI-stained images (magnification, ×400). (C) Proteoglycan content determined by dimethylmethylene blue (DMMB) assay [*P<0.05 compared to the lipopolysaccharide (LPS)-stimulated group, **P<0.01 compared to the LPS-stimulated group]. (D) Quantitative analysis of collagen-II.expression using IOD (***P<0.001 compared to the LPS-stimulated group). (E) Cell viability analyzed by NBT/DA PI staining (P>0.05).

